# Safety, effectiveness, and pharmacokinetics of adalimumab in children with polyarticular juvenile idiopathic arthritis aged 2 to 4 years

**DOI:** 10.1007/s10067-014-2498-1

**Published:** 2014-02-02

**Authors:** Daniel J. Kingsbury, Brigitte Bader-Meunier, Gina Patel, Vipin Arora, Jasmina Kalabic, Hartmut Kupper

**Affiliations:** 1 Randall Children’s Hospital at Legacy Emanuel, 501 N Graham, Suite 355, Portland, OR 97227 USA; 2 Hôpital Necker-Enfants Malades, Paris, France; 3 AbbVie, North Chicago, IL USA; 4 AbbVie Deutschland GmbH & Co. KG, Ludwigshafen, Germany

**Keywords:** Adalimumab, Polyarticular juvenile idiopathic arthritis, Safety

## Abstract

The objective of this study was to assess the safety of adalimumab in patients aged 2 to <4 years old or ≥4 years old weighing <15 kg with moderately to severely active polyarticular juvenile idiopathic arthritis (JIA). Clinical effectiveness and pharmacokinetics (PK) of adalimumab were also evaluated. This was an international, multicenter, open-label, phase 3b study in 32 patients with active JIA that were treated with adalimumab 24 mg/m^2^ (maximum = 20 mg/dose) every other week up to 120 weeks, with or without concomitant methotrexate. Adverse events (AEs) were summarized for completed visits. Efficacy endpoints included American College of Rheumatology pediatric (PedACR) 30/50/70/90 responses and JIA core components. Adalimumab serum trough concentrations were measured in a subset of patients. Among the patients, 88 % were female. Baseline mean age, weight, and JIA duration were 3 years, 13 kg, and 12 months, respectively; 39 % had elevated C-reactive protein. AE incidence rates included any AEs (29/32, 91 %), serious AEs (5/32, 16 %), infectious AEs (25/32, 78 %), and serious infections (3/32, 9 %). No deaths, malignancies, or opportunistic infections were reported. Growth was not adversely impacted. At week 96, 92 % of patients achieved PedACR30, and 77 % achieved PedACR70. Improvements in JIA core components were observed. Mean steady-state serum adalimumab trough concentrations were 7–8 μg/mL at weeks 12 and 24. Adalimumab was well tolerated in JIA patients aged 2 to <4 years old or ≥4 years old weighing <15 kg. The efficacy and PK of adalimumab were comparable to those seen in older JIA patients.

## Introduction

Juvenile idiopathic arthritis (JIA), the most common rheumatic disease of childhood, comprises a group of autoimmune diseases that often persists into adulthood with the potential for generating significant disability and growth impairment [1]. JIA has an estimated incidence of 15 per 100,000 and is 2.5 times more common in female patients [2, 3]. For the subset diagnosed with polyarticular onset/course JIA, defined as arthritis affecting ≥5 joints, the age of onset has a bimodal distribution, with peak incidences at 2–4 years and 10–14 years [2].

The antimetabolic agent, methotrexate (MTX), is commonly used in the treatment of polyarticular JIA; however, not all patients respond sufficiently to MTX, and some are intolerant of its side effects. The newer biologic agents, such as tumor necrosis factor (TNF) inhibitors, represent an advancement in the management of JIA, particularly for children who cannot achieve adequate disease control with traditional antirheumatic treatments; however, the effects of these agents in very young children with JIA (age <4 years) are not well understood.

Adalimumab is a fully human anti-TNF antibody that is approved for use in moderate to severe polyarticular JIA in patients ≥4 years of age in the US, EU, and Japan [4–6], and as of February 2013, adalimumab was also approved in EU for use in patients aged 2 to <4 years old [6]. Adalimumab has been shown to be safe and effective in JIA patients aged 4–17 years when dosed every other week (eow), [7] and in an international trial, clinical responses with adalimumab were maintained for up to 6 years [8]. Similar results were observed in a pediatric Japanese JIA population through 60 weeks of treatment [9]. However, adalimumab has not been systematically studied in patients <4 years of age, and limited data are available for patients ≥4 years of age who weigh <15 kg.

This study examined the safety of adalimumab in a very young JIA population with active polyarticular disease. Patients could be enrolled with or without concurrent MTX use and were to receive adalimumab for a minimum of 24 weeks. The primary objective of this report is to summarize the safety of adalimumab in this population over the course of the study; secondary objectives include analysis of clinical effectiveness and pharmacokinetic data.

## Patients and methods

### Patients

Eligible patients were aged 2 to <4 years or aged ≥4 years and weighing <15 kg with moderately to severely active polyarticular or polyarticular course JIA, as defined per the International League of Associations for Rheumatology (ILAR) criteria. Patients had moderately to severely active disease with ≥5 active joints at the time of study entry. In addition, in EU, patients must have previously failed, had an insufficient response to, or been intolerant of at least one DMARD, consistent with the local prescribing information for adalimumab in older children. Main study exclusion criteria were prior exposure to a TNF inhibitor or other biologic therapy, joint surgery within 2 months of screening (of joints to be assessed within the study), chronic recurring infection or active tuberculosis (TB), or significant concomitant illness. A parent or legal guardian provided written informed consent before any study procedures were performed.

### Study design

This was an international, multicenter, open-label, phase 3b study (trial registration: NCT00775437) conducted at 14 sites in the US and the EU. A central institutional review board or independent ethics committee approved the study at each site; the study was conducted in accordance with the International Conference on Harmonization good clinical practices and the Declaration of Helsinki. Prior to any study-related procedures, the informed consent statement was reviewed, signed, and dated by the patient’s parent. The size of the study was determined based upon a commitment to the US Food and Drug Administration (FDA) to provide safety and efficacy data for adalimumab when used in 2 to <4 year old patients with JIA, and, in accordance with the FDA, the proposed sample size for this study was approximately 30 patients. Qualified patients received adalimumab subcutaneously (24 mg/m^2^, maximum 20 mg/dose) eow for a minimum of 24 weeks in a clinical setting. The continuation of prior JIA treatments, such as MTX, stable doses of nonsteroidal anti‐inflammatory drugs (NSAIDs) and/or low-dose corticosteroids (equal to prednisone ≤0.2 mg/kg/day) was permitted. In the US, patients could continue in the study until reaching age 4 and ≥15 kg; in EU, patients could continue in the study for up to 1 additional year after reaching age 4 and ≥15 kg to allow time to transition to an appropriate treatment. Upon completion of the study, patients who transitioned to commercially available adalimumab were offered the chance to participate in a long-term JIA registry (STRIVE, NCT00783510). Patients had a screening visit, baseline visit, and visits at weeks 2, 4, 8, 12, 16, 20, and 24. Visits occurred every 12 weeks for patients who continued in the study after week 24.

### Safety

Adverse events (AEs), serious adverse events (SAEs), and events of special interest were collected throughout the study. Severe adverse events were events that caused considerable interference with the patient’s usual activities and may be incapacitating or life-threatening, though not considered an SAE. In contrast, SAEs include those that met any of the following criteria: death, life-threatening (i.e., would have resulted in immediate fatality without medical intervention), hospitalization, prolongation of hospitalization, persistent or significant disability, or any important medical event requiring medical or surgical intervention to prevent serious outcome. Patients were also monitored for SAEs/AEs for up to 70 days after their last dose of study medication or until they began commercial adalimumab, whichever came first. Laboratory assessments for chemistry, hematology, and serology were taken at baseline, weeks 12, 24, and every 12 weeks thereafter, including the final study visit.

### Efficacy measures

As secondary study endpoints, American College of Rheumatology pediatric (PedACR) 30/50/70/90 responses were determined at each visit, defined as at least 30 %, 50 %, 70 %, and 90 % improvement, respectively, from baseline in any three of the six following JIA core set variables, with worsening of ≥30 % in not more than one of the criteria: (1) Physician’s Global Assessment of Disease Activity (PhGA; 0–100 mm visual analog scale [VAS]); (2) Parent’s Global Assessment of Disease Activity (PaGA; 0–100 mm VAS); (3) functional ability as measured by the Disability Index of the Childhood Health Assessment Questionnaire (DI-CHAQ); (4) number of joints with active arthritis (AJC) in 73 joints; (5) number of joints with limitation of motion (LOM) in 69 joints; and (6) laboratory marker of inflammation, C-reactive protein (CRP) level. Other joint assessments included tender joint count (TJC) in 75 joints, swollen joint count (SJC) in 66 joints, and pain on passive motion (POM) in 75 joints. Parent’s Global Assessment of Pain (PaGA of Pain [0–100 mm VAS]) was also summarized. Patient height and weight were measured at baseline and at specified study visits. Additionally, patients were grouped by baseline height into four categories: <5th, ≥5th – <25th, ≥25th – <50th, and ≥50th percentile based on the US Centers for Disease Control and Prevention (CDC) growth charts. Mean CDC percentile changes in height and weight were calculated through 96 weeks. Patients may also have been evaluated for inactive disease, defined by the following criteria: no active arthritis, fever, rash, serositis, splenomegaly, generalized lymphadenopathy attributable to JIA, or JIA-associated uveitis, in addition to normal CRP level and PhGA indicating clinical disease quiescence.

### Pharmacokinetics

Per protocol, blood samples were provided by a subset of the study population (*n* = 15) to be analyzed for serum adalimumab concentrations and anti-adalimumab antibodies (AAA) at baseline and weeks 12 and 24 using a validated enzyme-linked immunosorbent assay.

### Statistical analyses

The intent-to-treat (ITT)/safety population included all patients who had received at least one dose of adalimumab. Safety data are summarized up to 120 weeks of treatment: AEs are reported as number and percentage of patients affected and as events per 100 patient-years (E/100 PYs). Efficacy data are presented based on observed values (weeks 12, 24, 60, and 96) and nonresponder imputation (NRI) (weeks 12 and 24). Growth data was analyzed as observed.

## Results

### Patient disposition

**Fig. 1:**
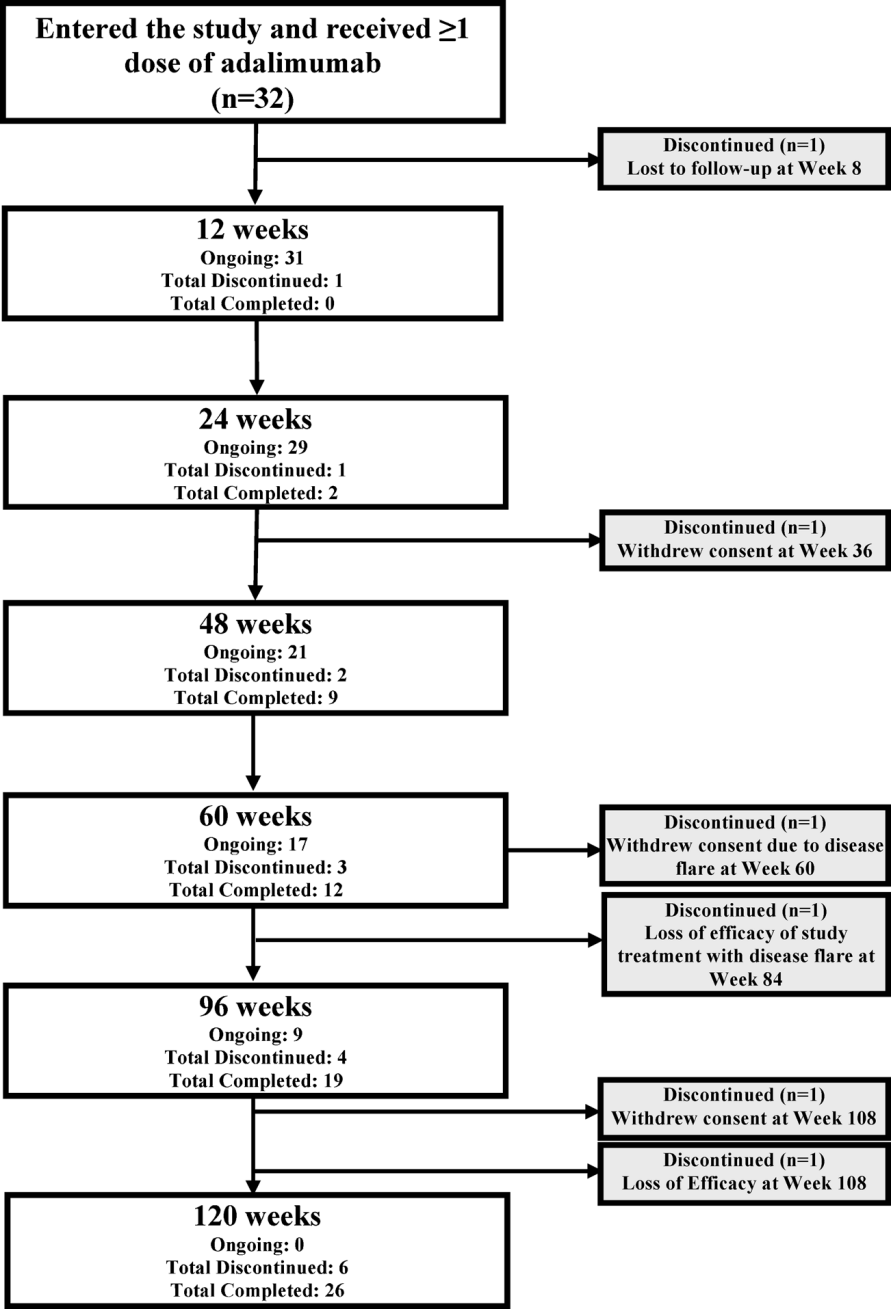
Patient disposition

A total of 32 patients were enrolled (15 from the US and 17 from the EU), and all received ≥1 dose of adalimumab, with 31 patients (97 %) completing 24 weeks of treatment (Fig. 1). Two patients withdrew prior to week 60, while one patient withdrew from the study at the week 60 visit. Three additional patients discontinued the study after week 60. Twenty-six patients (81 %) completed the study after achieving age and weight termination criteria, with 13 patients completing week 96 and three patients completing week 120.

### Baseline demographics, disease characteristics, and prior and concomitant therapies

Most patients were female and white (Table 1). The mean age at baseline was 3 years, with a mean JIA disease duration of 12 months. Baseline disease activity was consistent with moderately to severely active polyarticular JIA, with a mean AJC of 10.0. At baseline, 39 % had elevated CRP (≥0.9 mg/dL), 18 patients were ANA positive, and one patient was rheumatoid factor positive (RF+). Baseline JIA onset types by ILAR criteria were RF-negative polyarthritis (20/32, 62.5 %), extended oligoarthritis (8/32, 25 %), systemic onset with polyarticular course arthritis (2/32, 6.3 %), RF +  polyarthritis, and undifferentiated arthritis (1/32, 3.1 %) each.

**Table 1:** Baseline demographics and disease characteristics, and prior and concomitant JIA therapies

	Adalimumab (*N* = 32)
Demographics	
Age, years	3.0 (0.72)
Female, *n* (%)	28 (87.5)
White, *n* (%)	25 (78.1)
Weight, kg	13.4 (2.0)
Height, cm	93.0 (5.8)
Duration of JIA, months	12.3 (9.3)
Disease characteristics	
PedACR core variables	
PhGA (VAS)	55.3 (19.70)
PaGA (VAS)	47.6 (25.91)
DI-CHAQ (0–3)	1.2 (0.66)
AJC73	10.0 (7.47)
LOM69	8.6 (7.69)
CRP, mg/dL^a^	1.6 (2.43)
Other variables	
CRP <0.9 mg/dL, *n* (%)^a^	19 (61.3)
ANA positive, *n* (%)	18 (56.3)
RF positive, *n* (%)	1 (3.1)
Prior JIA therapies	
Methotrexate, *n* (%)	25 (78.1)
NSAIDs, *n* (%)	20 (62.5)
Systemic corticosteroids, *n* (%)	22 (68.8)
Concomitant JIA therapies	
Methotrexate, *n* (%)	27 (84.4)
NSAIDs, *n* (%)	18 (56.3)
Systemic corticosteroids, *n* (%)^b^	20 (62.5)

All values are expressed as the mean (standard deviation), unless indicated otherwise. ^a^ *n* = 31. ^b^Systemic corticosteroids include oral, injected, and rectal preparations and do not include nonsystemic preparations (ophthalmologicals, dermatologicals, and inhalants)

*AJC* active joint count, *CRP* C-reactive protein (<0.9 mg/dL = normal), *DI-CHAQ* Disability Index Childhood Health Assessment Questionnaire, *JIA* juvenile idiopathic arthritis, *LOM* limitation of motion, *NSAID* nonsteroidal anti-inflammatory drug, *PaGA* Parent’s Global Assessment, *PhGA* Physician’s Global Assessment, *RF* rheumatoid factor, *VAS* visual analog scale of 0–100 mm

Most patients (25/32, 78 %) had received MTX as a prior JIA therapy. The majority of patients (27/32, 84 %) took concomitant MTX during the study; of these, 59 % received concomitant folic acid. Only one patient (3 %) took concomitant hydroxychloroquine, while the remainder of the patients did not receive concomitant DMARDs. Approximately 63 % of patients reported use of systemic corticosteroids for their JIA, and 56 % reported use of NSAIDs during the study. A total of 31 patients (97 %) took at least one non-rheumatic medication during the study, with folic acid, standard childhood vaccines, paracetamol, and amoxicillin being reported most frequently.

### Safety

Safety data are summarized for all patients up to 120 weeks of treatment. Mean duration (SD) of adalimumab exposure was 515 (245) days; all subjects had at least 57 days of adalimumab exposure, with a maximum exposure of 910 days. This analysis represents approximately 45.1 PYs of exposure to adalimumab.

A total of 29 patients (90.6 %) reported at least one AE (Table 2). The most frequently reported AEs (occurring in ≥10 % of patients) were nasopharyngitis (*n* = 8, 25 %), pyrexia (*n* = 7, 22 %), bronchitis, cough, rhinorrhea, and upper respiratory infection (*n* = 6 each, 19 %), otitis media, worsening of rheumatoid arthritis (JIA), and vomiting (*n* = 5 each, 16 %), and diarrhea, gastroenteritis, rash, and rhinitis (*n* = 4 each, 13 %), regardless of investigator’s assessment for any relationship to study drug. Two patients discontinued the study due to an AE (worsening of JIA and loss of efficacy with JIA flare). While a majority of patients had nonserious events, five patients were reported to have one SAE each, which included dental caries, gastroenteritis rotavirus, worsening of JIA, type 1 diabetes mellitus, and varicella; these were considered to be “not related” or “probably not related” to adalimumab by the investigators. All SAEs were considered to be mild or moderate with the exception of diabetes mellitus (new onset, type 1), which was considered to be severe.

**Table 2:** Overview of adverse events (AEs)

	*N* = 32	PYs = 45.1
	*n* (%)	E (E/100 PYs)
Any AE	29 (90.6)	217 (481)
At least “possibly drug related” per the investigator	11 (34.4)	22 (48.8)
Severe AE	6 (18.8)	6 (13.3)
Serious AE^a^	5 (15.6)	5 (11.1)
AE leading to discontinuation of study drug^b^	2 (6.3)	2 (4.4)
Infectious AE^c^	25 (78.1)	93 (206)
Serious infectious AE^d^	3 (9.4)	3 (6.7)
Injection site-related AE	4 (12.5)	6 (13.3)

*E* number of events, *PYs* patient-years. ^a^Serious AEs included dental caries, gastroenteritis rotavirus, worsening of JIA, type 1 diabetes mellitus, and varicella. ^b^AEs leading to discontinuation of study drug were worsening of JIA and JIA flare. ^c^No opportunistic infections or cases of TB were reported. ^d^Three patients had infections that were categorized as serious (one case each of dental caries, rotavirus gastroenteritis, and varicella virus)

Regarding AEs of special interest with respect to TNF blockade, there were no cases of congestive heart failure-related AEs, demyelinating disease, lupus-like syndrome, malignancies, opportunistic infections/TB, blood dyscrasias, or deaths. The only AEs of special interest reported in this study were infections (serious and nonserious) and injection site-related AEs. The most frequently reported infections (≥10 % of patients) were nasopharyngitis, upper respiratory tract infection, bronchitis, otitis media, rhinitis, and gastroenteritis. Most infections were mild or moderate; one patient was reported to have a severe event (otitis media). All but five patients had infections that were considered to be “not related” or “probably not related” to adalimumab by the investigator. In these five patients, bronchitis, cystitis, ear infection, laryngitis, otitis media, pharyngitis, pneumonia, and viral pharyngitis were deemed as “possibly” or “probably related” to study drug by the investigator. Three patients had infections that were reported as serious (one case each of dental caries, gastroenteritis rotavirus, and varicella). All three serious infections were considered by the investigator as “not related” to adalimumab and were mild or moderate in severity; all three subjects continued their participation in the study. Injection site-related events considered “probably related” to adalimumab were reported for four patients (injection site pain, injection site pruritus and swelling, injection site rash, and unspecified injection site reaction) but were considered to be mild and did not affect study participation.

**Fig. 2:**
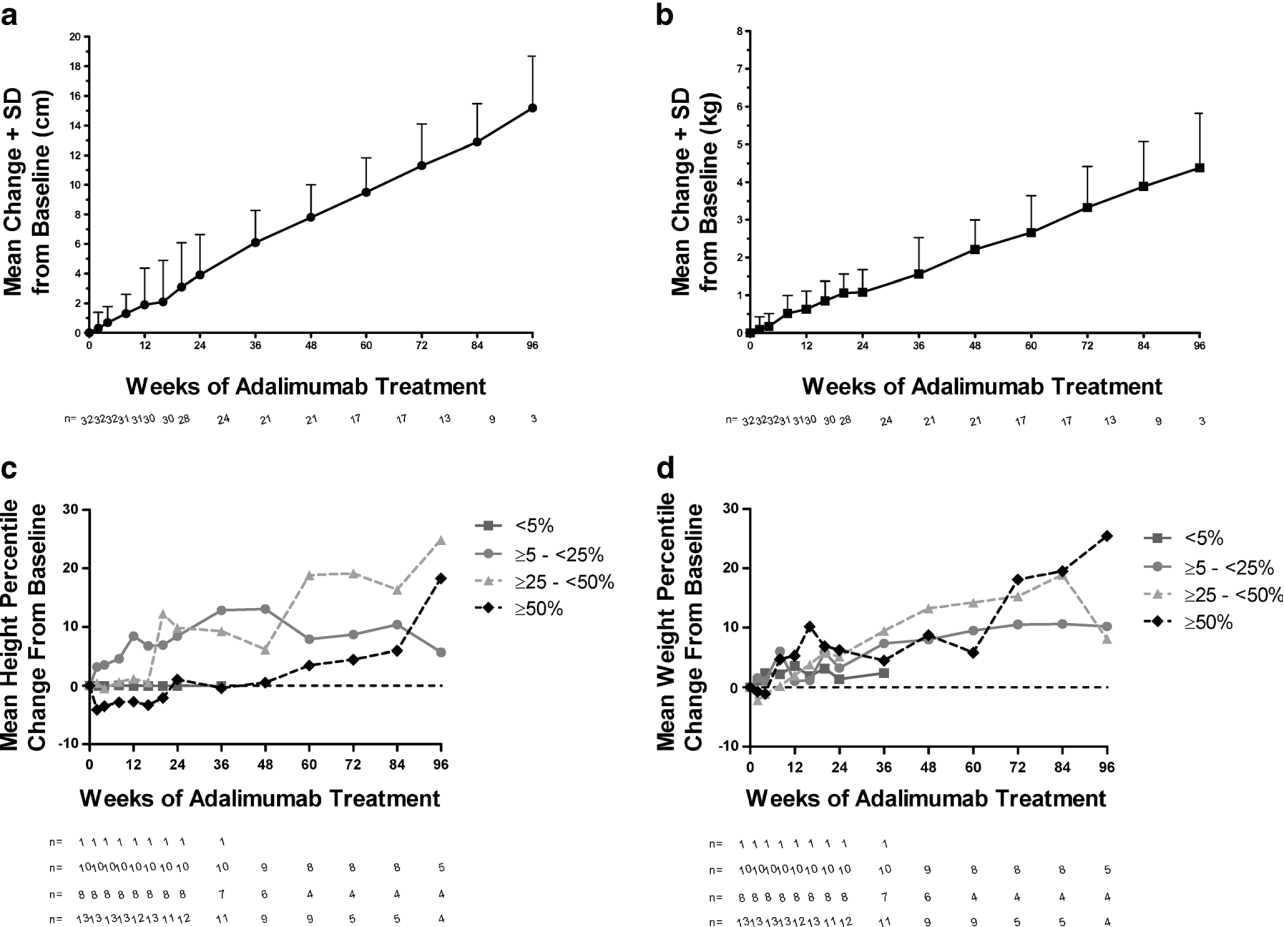
Mean change from baseline in growth over time. **a** Mean change in height + standard deviation (SD) in centimeters (cm) over 96 weeks of adalimumab treatment. **b** Mean change in weight + standard deviation (SD) in kilograms (kg) over 96 weeks of adalimumab treatment. **c** Mean height percentile change from baseline over 96 weeks of adalimumab treatment in <5th, ≥5th – <25th, ≥25th – <50th, and ≥50th percentile based on the US Centers for Disease Control and Prevention (CDC) growth charts. **d** Mean weight percentile change from baseline over 96 weeks of adalimumab treatment in <5th, ≥5th – <25th, ≥25th – <50th, and ≥50th percentile based on the US Centers for Disease Control and Prevention (CDC) growth charts. Observed analysis

Growth, as measured by height and weight, was not adversely affected by adalimumab treatment in these children (Fig. 2). Mean change (SD) from baseline to week 60 was +9.5 cm (2.34) for height (Fig. 2a) and +2.66 kg (0.98) for weight (Fig. 2b) and at week 96 was +15.2 cm (3.48) for height and +4.38 kg (1.44) for weight. Among the 32 patients, only one (3.1 %) was in the <5th percentile based on the CDC growth charts and had a −0.03 % and 2.4 % change in mean height and weight percentiles, respectively. Patients had a 5.6, 24.9, and 18.2 % change in mean height percentile for ≥5th – <25th, ≥25th – <50th, and ≥50th percentile, respectively, through 96 weeks of ADA treatment (Fig. 2c). Similarly, there was a 10.2, 8.1, and 25.5 % change in mean weight percentile for ≥5th – <25th, ≥25th – <50th, and ≥50th percentile, respectively (Fig. 2d).

### Efficacy

**Fig. 3:**
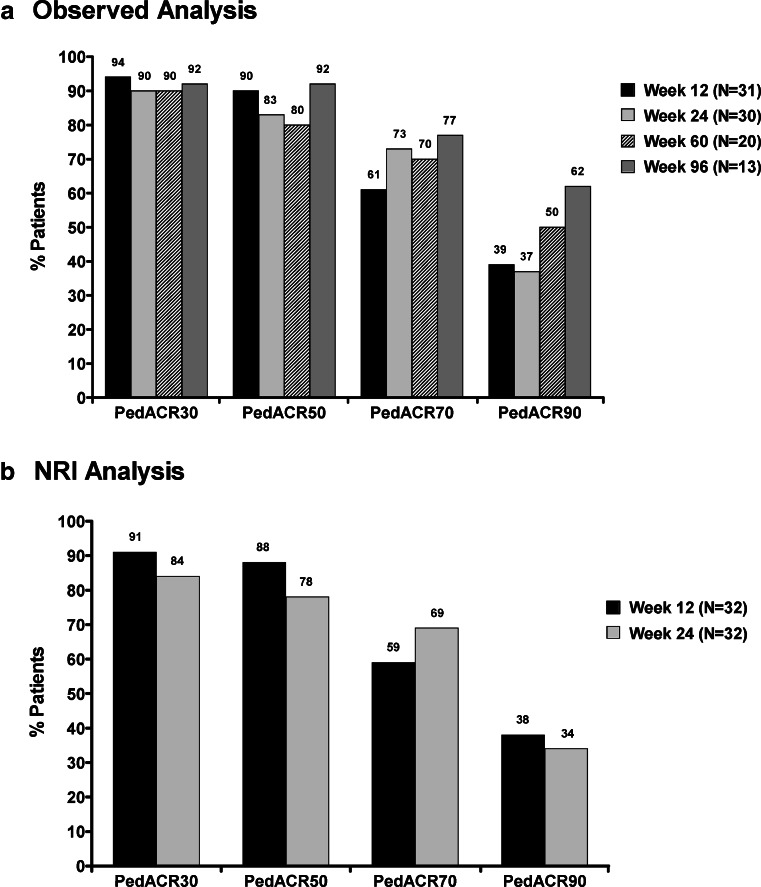
PedACR 30/50/70/90 response rates over time. **a** Percentage of patients achieving PedACR 30/50/70/90 responses at weeks 12, 24, 60, and 96. Observed analysis. **b** Percentage of patients achieving PedACR 30/50/70/90 responses at weeks 12 and 24. Nonresponder imputation (NRI)

The percentage of patients with PedACR30 responses at weeks 12, 24, 60, and 96 were 94 %, 90 %, 90 %, and 92 %, respectively (Fig. 3a); PedACR50/70/90 responses at weeks 12, 24, 60, and 96 were 90 %/61 %/39 %, 83 %/73 %/37 %, 80 %/70 %/50 %, and 92 %/77 %/62 %, respectively. Additionally, all three subjects completing week 120 had a good and sustained response achieving PedACR90. PedACR response rates at weeks 12 and 24, using NRI method, were similar to rates in the observed case analysis (Fig. 3b).

Statistically significant improvements from baseline in the JIA core variables (PhGA, PaGA, DI-CHAQ, AJC, and LOM), joint assessments (TJC75, SJC66, and POM75), and PaGA of pain were observed with adalimumab treatment at weeks 12, 24, 60, and 96 weeks (Table 3). Mean CRP improved from baseline to weeks 12, 24, and 60 but did not achieve statistical significance.

**Table 3:** JIA outcomes at weeks 12, 24, 60, and 96 (observed analysis)

	Baseline (*N* = 32)	Week 12 change from baseline (*n* = 31)	Week 24 change from baseline (*n* = 30)	Week 60 change from baseline (*n* = 20)	Week 96 change from baseline (*n* = 13)
JIA core variables					
PhGA (VAS)	55.3 (19.70)	−41.4 (21.20)*	−45.3 (21.32)*	−42.7 (28.17)*	−47.5 (24.42)*
PaGA (VAS)	47.6 (25.91)	−28.1 (29.91)*	−32.2 (29.74)*	−34.5 (33.31)*	−45.8 (29.10)*
DI-CHAQ (0–3)	1.2 (0.66)	−0.5 (0.64)**	−0.5 (0.69)**	−0.6 (0.71)**	−0.8 (0.56)*
AJC73	10.0 (7.47)	−7.3 (4.52)*	−7.2 (5.60)*	−9.5 (7.50)*	−8.9 (7.04)*
LOM69	8.6 (7.69)	−5.6 (4.80)*	−5.6 (5.54)*	−5.5 (8.31)**	−7.5 (6.73)**
CRP, mg/dL^a^	1.6 (2.43)	−0.6 (2.65)	−0.2 (3.20)	−0.3 (1.83)	0.1 (1.60)
Other assessments					
TJC75	3.8 (5.02)	−2.7 (5.09)**	−3.0 (5.54)**	−4.5 (5.85)**	−4.0 (5.46)**
SJC66	8.9 (7.37)	−6.2 (4.24)*	−6.3 (5.83)*	−8.4 (7.15)*	−8.5 (6.89)*
POM75	5.3 (4.66)	−4.9 (4.59)*	−4.1 (7.32)**	−5.9 (5.25)*	−5.7 (5.12)**
PaGA of pain (VAS)	46.1 (25.73)	−27.2 (25.36)*	−29.5 (28.28)*	−35.2 (34.40)*	−45.1 (26.45)*

*AJC* active joint count, *CRP* C-reactive protein, *DI-CHAQ* Disability Index Childhood Health Assessment Questionnaire, *LOM* limitation of motion, *PaGA* Parent’s Global Assessment, *PhGA* Physician’s Global Assessment, *POM* pain on motion, *SJC* swollen joint count, *TJC* tender joint count, *VAS* visual analog scale of 0–100 mm

*P < 0.001; **P < 0.05. The *t* test was used to calculate statistical significance, and all values are expressed as the mean (standard deviation)

^a^ *n* = 31 at baseline, *n* = 28 at weeks 12 and 24, *n* = 20 at week 60, and *n* = 12 at week 96

### Pharmacokinetics

Mean steady-state serum adalimumab trough concentrations were measured at weeks 12 and 24 and were found to be consistent with levels achieved in older children (age 4–17) with JIA (7). In the current study, adalimumab trough concentrations were noted to be numerically higher in patients who received concomitant MTX (7–8 μg/mL) compared to those who did not (6 μg/mL), but there were too few patients (*n* = 4) in the non-MTX group to allow statistical analysis. In addition, correlation of pharmacokinetics with efficacy data could not be performed due to the small sample size. Among the 15 patients who had samples for pharmacokinetic analysis, only one patient (6.7 %) developed AAA+ during the 24 weeks that were evaluated.

## Discussion

Treatment with adalimumab has been shown to significantly improve the signs and symptoms of active polyarticular JIA in patients aged 4 to 17 years [7, 9]. Limited data are available on the use of TNF inhibitors in children with JIA who are younger than 4 years of age, and no previous studies have evaluated the safety of adalimumab in this population. The objectives of this phase 3b open-label study were to systematically evaluate the safety, effectiveness, and pharmacokinetics of adalimumab in children with JIA aged 2 to <4 years or ≥4 years weighing <15 kg. Overall, adalimumab was well tolerated and efficacious in this very young patient population.

Regarding the safety profile, infections were the most commonly reported AEs. No cases of opportunistic infection or TB occurred in up to 120 weeks of adalimumab treatment, but one SAE of varicella infection was reported, consistent with incidence data from other studies [10, 11]. New onset type I diabetes mellitus developed in one patient and was reported as an SAE, but considered “not related” to study drug by the investigator. Although a relationship between adalimumab and type I diabetes mellitus cannot be excluded [12–14], the patient’s father also has type I diabetes mellitus, possibly providing the basis for additional genetic susceptibility for diabetes mellitus in the patient. Additionally, it is well known that there are increased risk and frequency of type I diabetes mellitus developing in patients with JIA [15, 16]. Another SAE, recorded as “not related” to study drug, involved a child with preexisting dental caries who required hospitalization for a sedated dental procedure. The prevalence of injection site reactions (4/32, 12.5 %) was notably lower than in previous literature. Although injection site reactions are not uncommon, the low number of reactions recorded in this study may be reflective of reporting practices more than event rates. During 45.1 PYs of adalimumab exposure, no patients were withdrawn due to an SAE; however, two patients discontinued the study due to nonserious AEs (“worsening of JIA” and “JIA flare”).

With respect to AEs of special interest, there were no reports of congestive heart failure-related AEs, demyelinating disease, lupus-like syndrome, malignancies, opportunistic infections/TB, allergic reactions, blood dyscrasias, or deaths. Moreover, no safety events were identified that were unique to this young population, and the findings were similar to those obtained in trials of older pediatric patients [7, 9] and in adults with rheumatoid arthritis (RA) [17]. The safety profile was, in fact, very comparable to another study by Imagawa et al. [9] that evaluated the efficacy and safety of adalimumab in patients aged 4–17 years with JIA in Japan. Six out of 25 patients (24 %) in the Japanese study had an SAE, compared to 15.6 % in this study. Similarly, 21 (84 %) versus 25 (78.1 %) had infections, and 3 (12 %) versus 3 (9.4 %) had reported serious infections in the Japanese study and our study, respectively.

Growth parameters are of particular interest in this very young patient population, given the potential negative impact of chronic, active JIA, and standard therapies (such as corticosteroids) on normal childhood development [18]. In this study, height and weight measurements over time revealed that treatment with adalimumab did not adversely impact growth in this age group.

Although not the primary endpoint, results of this study also demonstrated the efficacy of adalimumab in improving clinical response and reducing disease activity in very young patients with JIA. A comparison of the PedACR response rates between the patients in this study and older children in prior JIA trials [7, 9] shows that similar, high rates of favorable clinical responses occurred in both age groups. Furthermore, treatment with adalimumab in all three of these JIA studies led to sustained improvement in JIA core components and other efficacy parameters. These findings are also consistent with those observed in adult RA studies [19, 20].

Serum adalimumab trough concentrations obtained in a subset of this study population showed that the mean steady-state adalimumab levels were within the range observed in children with JIA aged 4 to 17 years [7] and were consistent with levels observed in other adalimumab trials [21, 22]. Although mean adalimumab levels were numerically higher in patients who received concomitant MTX therapy, the small number of patients in the non-MTX arm (*n* = 4) makes it difficult to draw conclusions regarding the impact of MTX on adalimumab concentrations in this population. Importantly, the results demonstrate that steady-state levels of adalimumab, considered to be therapeutic, can be achieved with or without MTX in this very young JIA population. Additional serologic testing identified only one of 15 patients (6.7 %) with AAA, and due to the very small sample size, no conclusions regarding the effect of AAA on safety or efficacy could be made.

Limitations of this study include the open-label nature, the small number of patients, the indirect measurement of pain (via parent assessment), the limited length of follow-up to date, which does not allow conclusions to be made about long-term side effects and safety, and lack of assurance that responders could continue receiving adalimumab upon completion of the study. In the US, patients could transition to commercially available adalimumab after the age of 4 and achieving weight ≥15 kg. In the EU, patients were allowed to continue study treatment with adalimumab for up to 1 additional year after achieving the age of 4 and ≥15 kg, providing time necessary to transition to medication until the local approval was obtained for treatment continuation. All patients continuing on commercial adalimumab after study completion were offered the opportunity to enroll into a long-term JIA registry, and 12 patients (37.5 %) transitioned from the study to the JIA registry. Even so, despite these limitations, the results described herein are in agreement with results obtained in other JIA studies of adalimumab safety and efficacy [7, 9].

In summary, the results suggest that adalimumab is a well-tolerated treatment option with or without concurrent MTX in patients with active polyarticular JIA who are aged 2 to <4 years or ≥4 years weighing <15 kg. No new safety signals occurred in this study. There were no events of opportunistic infections/TB, malignancies, or deaths reported, and the safety of adalimumab in this patient population was comparable to that observed in older JIA patients. Adalimumab was also efficacious in this younger patient population, resulting in high and sustained clinical responses for up to 96 weeks. Taken together, these results provide the first clinical trial data on the effects of adalimumab for the treatment of moderately to severely active JIA in this very young population.

## Key points

- Adalimumab therapy with or without methotrexate was safe and well tolerated in this very young population, with outcomes comparable to those observed in older JIA patients treated with adalimumab
- Adalimumab proved efficacious with sustained improvement of core JIA disease activity parameters for 96 weeks of therapy
- These novel data represent the first systematic clinical trial results reported for adalimumab therapy in this very young population with JIA, a group with significant unmet medical need
